# Optimized Phosphorus Application Enhances Wheat Stem Lodging Resistance Under Spring Low-Temperature Stress

**DOI:** 10.3390/plants13212980

**Published:** 2024-10-25

**Authors:** Xiang Chen, Qianqian Liu, Baoqiang Zheng, Jincai Li

**Affiliations:** 1 College of Agriculture, Anhui Agricultural University, Hefei 230036, China; cx2468@ahau.edu.cn (X.C.); 15855105367@stu.ahau.edu.cn (Q.L.); zhengbaoqiang@ahau.edu.cn (B.Z.); 2 Jiangsu Collaborative Innovation Centre for Modern Crop Production, Nanjing 210095, China

**Keywords:** *Tritium aestivum* L., cold stress, base stem characteristics, phosphorus fertilizer postpone, lodging resistance index, yield

## Abstract

Spring low-temperature stress (LTS) has become a major limiting factor for the development of high yield, quality and efficiency in wheat production. It not only affects the function of wheat leaves and the development of spikes but also impacts stem lodging resistance, and may experience elevated risk of stem lodging. This study conducted a field pot experiment to assess the effect of phosphorus fertilizer application mode on wheat stem lodging resistance under spring LTS. Two wheat varieties, Yannong19 (YN19, cold-tolerant variety) and Xinmai26 (XM26, cold-sensitive variety) used as the experiment material. Two phosphorus fertilizer application models including traditional phosphorus application (TPA) and optimized phosphorus application (OPA) were employed. Temperature treatment was conducted at 15 °C (CK) and −4 °C (LT) in a controlled phytotron. Our results showed that spring LTS decreased the stem wall thickness and internode fullness, and altered stem anatomical structure and chemical composition, resulting in a decrease in wheat stem mechanical strength and lodging resistant index. Compared with TPA, the OPA increased the stem wall thickness and internode fullness. The thickness of the stem mechanic tissue layer and parenchymatous tissue, and the area of the large vascular bundle and small vascular bundle were increased by the OPA, which alleviated the damage to stem cell walls caused by spring LTS. At the same time, the OPA also increased the contents of lignin, cellulose, and soluble sugar, improving the C/N ratio in wheat stem. Due to the improved stem morphological characteristics, anatomical structure, and chemical compositions, the wheat stem exhibited enhanced lodging resistance, which increased the lodging resistant index of the 2nd and 3rd internodes of YN19 and XM26 by 27.27%, 11.63% and 14.15%, 15.73% at the dough stage compared with TPA under spring LTS. Meanwhile, OPA could not only alleviate the yield loss caused by spring LTS, but also increase the grain yield without spring LTS. This study indicated that OPA enhances wheat stem lodging resistance under spring LTS, and would be meaningful and practical for improving wheat resistance to low-temperature stress.

## 1. Introduction

Wheat (*Tritium aestivum* L.) is not only one of the world’s three major food crops, but also a key staple crop in China [1]. According to data from the National Bureau of Statistics (NBS) of China, in 2023, the cultivation area and yield of wheat in China were 23,627.2 thousand hectares and 136,590 thousand tons, accounting for approximately 19.86% and 19.64% of the total cultivation area and yield of grain crops [2]. Therefore, the safe production of wheat is of great significance for ensuring national food security, promoting socio-economic development, and improving people’s quality of life. The Sixth Assessment Report of the Intergovernmental Panel on Climate Change (IPCC) reported that global surface temperatures have risen by approximately 0.8–1.3 °C from 1850 to 1900 compared to that in 2010 to 2019, and is expected to rise exceed 1.5 °C in the future [3]. The ongoing global climate warming poses major challenges for food production and adverse climate change-related events such as low temperatures, high temperatures, strong winds, and heavy rainfall are becoming increasingly frequent [4]. Among them, low-temperature stress (LTS) poses a great threat to the production of major wheat-producing countries and regions such as China [5], Australia [6], Europe [7], and the United States [8]. The Huang-Huai region is the largest wheat-producing area in China. Global warming has accelerated the growth of winter wheat, advancing the critical growth period (jointing and booting), and increasing its vulnerability and susceptibility to damage from spring LTS [9,10,11]. Therefore, spring LTS has become one of the important factors limiting the high yield, high quality, and efficient development in the Huang-Huai region [12].

Spring LTS in the Huang-Huai wheat region often occurs from March to April [13], which means that the temperature in the season of rebirth and growth rises faster, and in the late spring the temperature is lower than normal [14]. The jointing to booting stage is a critical period of simultaneous nutritional and reproductive growth of wheat. Meanwhile, the wheat young spike is in a key period of differentiation and development, and is highly sensitive to LTS [12]. In this stage, if encountering spring LTS, causing damage or death to the main stem, large tillers, and young spike, resulting in spikelets or whole spike infertile. Generally, mild to moderate spring LTS leads to a 10% to 30% reduction in wheat yield, while severe spring LTS can result in a reduction of over 50% in wheat yield and deterioration of grain quality [15,16,17].

Regarding the disaster mechanism of wheat spring LTS, previous studies have conducted extensive research from the perspective of source-sink organs. The studies by Zhang et al. [5] and Liu et al. [18] showed that spring LTS not only damages the integrity of the functional leaf mesophyll cell structure of wheat, but also leads to an imbalance in reactive oxygen species (ROS) metabolism in the leaves, and an increase in malondialdehyde (MDA) content leads to a decrease in its photosynthetic performance. Ke et al. [19] found that spring LTS can affect the accumulation and distribution of dry matter in wheat plants, especially decreasing the transport and distribution of photosynthetic compounds to spike. Currently, further research by Lin et al. [20] showed that the accumulation of assimilates in wheat spikes and the decrease in their allocation to different spike positions under spring LTS could restrain spike and floret development, affecting the grain number at different spike positions and resulting in a significant decrease in wheat yield. However, previous studies mostly focus on leaves or spikes, and there are few reports on the effects of spring LTS on wheat stem lodging resistance. Wheat stems have the functions of supporting aboveground plants, transporting and storing nutrients. Global climate change has led to frequent occurrences of extreme weather such as strong winds and heavy rainfall during the later stages of wheat growth, resulting in frequent lodging phenomena and yield losses of up to 10–30% [21]. For example, in 2024, some wheat fields experienced lodging due to the large plant density and strong winds in the middle and later stages of wheat growth, seriously affecting the yield and quality of wheat [22].

Phosphorus, as the most important nutrient element in crop nutrition after nitrogen, is a component of various compounds such as nucleic acids, phospholipids, adenosine triphosphate, and plays an important role in various metabolic activities [23,24]. However, in natural ecosystems, the phosphorus that is lost from the soil-plant cycling system has to be replaced by the slow process of rock weathering or added via fertilizer in human-managed systems, and approximately 40% of the global agricultural lands are deficient in phosphorus [25,26]. Therefore, improving the efficiency of phosphorus fertilizer utilization in agricultural production is a necessary measure for sustainable agricultural development. Many studies have shown that the rational application of phosphorus fertilizer was able to improve the lodging resistance of crop stems [27,28,29]. Deng et al. [27] showed that the stem diameter and stalk strength of Quinoa applied with 100 kg ha^−1^ phosphorus was increased by 9.5% and 23.2%, and the lodging rate was decreased by 44.5% in a 2-year field experiment, compared with CK. Liang et al. [28] found that the amount of phosphorus applied is closely related to the lodging resistance of maize and optimized phosphorus application (OPA) could promote the accumulation of dry matter between maize internodes, change the ear position coefficient, and improve lodging resistance. The study by Xiang et al. [29] also showed that OPA can improve the thickness, C/N ratio, cell wall cellulose and lignin content, mechanical strength, and lodging resistance index of intercropping soybean stem internodes, enhancing the lodging resistance of soybean. Previous studies have found that optimized phosphorus application could alleviate the damage of spring LTS by improving the root physiology, photosynthesis, spike development and setting [30,31,32]. However, there have been no reports on the effect of OPA on the lodging resistance of wheat stems under the spring LTS.

In this study, a controlled phytotron experiment was conducted with two contrasting wheat varieties with different cold sensitivities under spring LTS (−4 °C for 4 h) and different phosphorus applications during the anther differentiation period. The objective was to (1) quantify the effects of OPA on the morphological characteristics, anatomical structure, and chemical composition of wheat stems under spring LTS, (2) assess the effects of OPA on the wheat stem lodging resistance under spring LTS, (3) analyze the effects of OPA on wheat yield under spring LTS. The results of this study could provide a theoretical basis for research and development of stress-resistant cultivation measures for wheat, and also be used to guide disaster prevention and reduction in wheat production.

## 2. Results

### 2.1. Stem Wall Thickness and Internode Fullness

**Figure 1:**
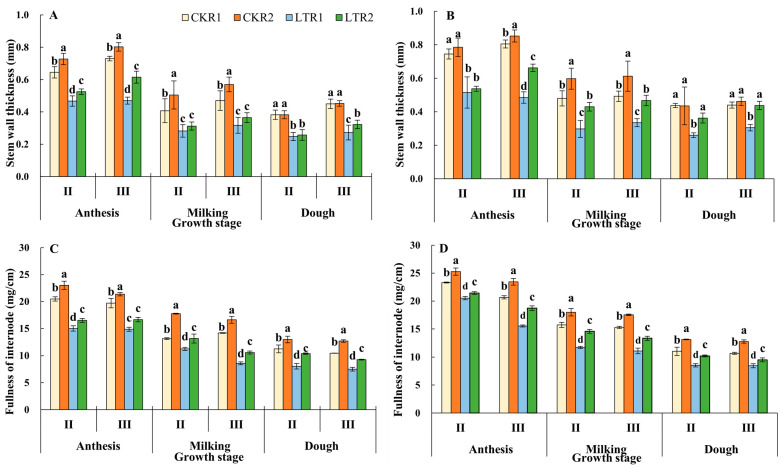
Effects of OPA on stem wall thickness and internode fullness of wheat under spring LTS. (**A**) Stem wall thickness of YN19. (**B**) Stem wall thickness of XM26. (**C**) Internode fullness of YN19. (**D**) Internode fullness of XM26. CKR1 and LTR1 represent the 15 °C and −4 °C under traditional phosphorus application, respectively. CKR2 and LTR2 represent the 15 °C and −4 °C under optimized phosphorus application, respectively. II and III indicate the second and third internode of the main stem. Values are mean ± standard deviation. Different lowercase letters above the bars indicate significant differences among treatments for the same cultivar at the 0.05 level.

The stem wall thickness and internode fullness of the second and third internode showed a decreasing trend in each treatment of the two varieties with the growth process moving forward (Figure 1). Compared with CKR1, the thickness of the 2nd and 3rd internodes of YN19 in LTR1 and LTR2 significantly decreased by 35.29%, 32.68% and 39.44%, 28.33% at the dough stage, respectively; meanwhile the thickness of the 2nd and 3rd internodes of XM26 in LTR1 and LTR2 decreased by 40.57%, 17.14% and 30.68%, 0.57%, respectively. Compared with the TPA at the same temperature levels, the thickness of the 2nd and 3rd internodes of YN19 and XM26 in LTR2 under OPA increased by 4.04%, 18.35% and 39.42%, 43.44%, respectively. At the dough stage in LTR1 and LTR2 treatments, the fullness of the 2nd and 3rd internodes decreased by 7.69%~28.32% and 11.14%~28.13% in YN19, and 7.36%~22.50% and 10.73%~20.66% in XM26, respectively, compared with CKR1; whereas the fullness of the 2nd and 3rd internodes of YN19 and XM26 in LTR2 under OPA increased by 28.78%, 23.63% and 19.54%, 12.51%, compared with LTR1, respectively. Therefore, spring LTS decreased the thickness of stem internodes and internode fullness, while OPA was beneficial for increasing the thickness of stem internodes and internode fullness under spring LTS.

### 2.2. Stem Anatomical Parameters

#### 2.2.1. Mechanic Tissue Layer Thickness and Parenchymatous Tissue Thickness

**Figure 2:**
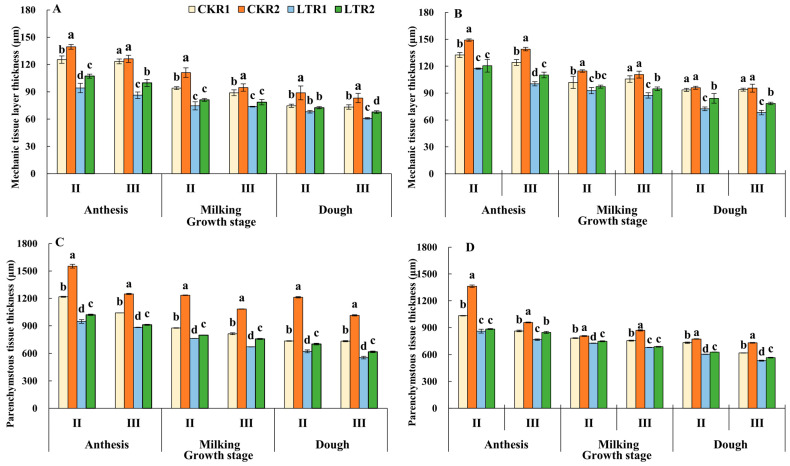
Effects of OPA on stem mechanic tissue layer thickness and parenchymatous tissue thickness of wheat under spring LTS. (**A**) Stem mechanic tissue layer thickness of YN19. (**B**) Stem mechanic tissue layer thickness of XM26. (**C**) Parenchymatous tissue thickness of YN19. (**D**) Parenchymatous tissue thickness of XM26. CKR1 and LTR1 represent the 15 °C and −4 °C under traditional phosphorus application, respectively. CKR2 and LTR2 represent the 15 °C and −4 °C under optimized phosphorus application, respectively. II and III indicate the second and third internode of the main stem. Values are mean ± standard deviation. Different lowercase letters above the bars indicate significant differences among treatments for the same cultivar at the 0.05 level.

With the advancement of the growth stage, the mechanic tissue layer thickness and parenchymatous tissue thickness of the 2nd and 3rd internodes of YN19 and XM26 showed a decreasing trend in each treatment from the anthesis to dough stage (Figure 2). Compared with CKR1, the mechanic tissue layer thickness and parenchymatous tissue thickness of the 3rd internodes of YN19 in the LTR1 and LTR2 treatment were decreased by 16.96%, 7.59% and 24.26%, 15.66% at the dough stage, respectively; whereas it increased by 11.28% and 11.35% in the treatments of LTR2, compared with LTR1. The mechanic tissue layer thickness and parenchymatous tissue thickness of the 3rd internodes of XM26 under TPA were decreased by 27.24% and 13.94% after spring LTS and increased by 14.93% and 6.36% under LTR2 treatments compared with LTR1. Additionally, the mechanic tissue layer thickness and parenchymatous tissue thickness of the 2nd internodes of the two varieties showed a similar trend of changes in the 3rd internode. Thus, spring LTS resulted in decreased mechanic tissue layer thickness and parenchymatous tissue thickness of the 2nd and 3rd in two varieties, and OPA treatment alleviated the damage of spring LTS.

#### 2.2.2. Large Vascular Bundle Area and Small Vascular Bundle Area

**Figure 3:**
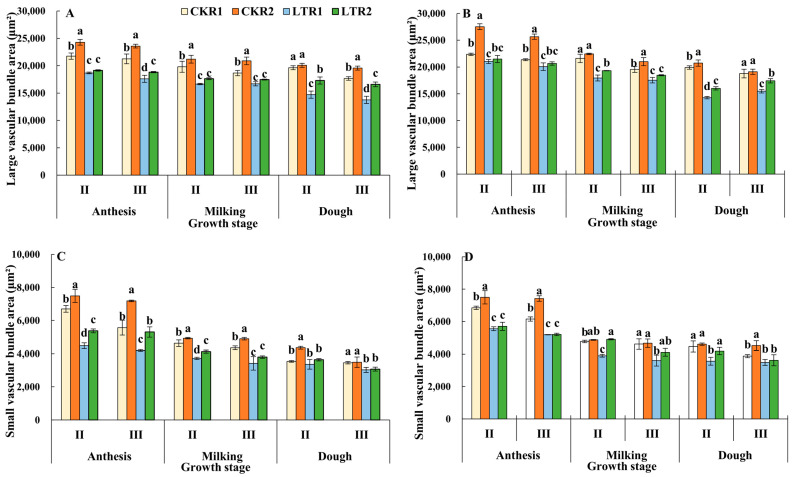
Effects of OPA on large vascular bundle area and small vascular bundle area of wheat under spring LTS. (**A**) Large vascular bundle area of YN19. (**B**) Large vascular bundle area of XM26. (**C**) Small vascular bundle area of YN19. (**D**) Small vascular bundle area of XM26. CKR1 and LTR1 represent the 15 °C and −4 °C under traditional phosphorus application, respectively. CKR2 and LTR2 represent the 15 °C and −4 °C under optimized phosphorus application, respectively. II and III indicate the second and third internode of the main stem. Values are mean ± standard deviation. Different lowercase letters above the bars indicate significant differences among treatments for the same cultivar at the 0.05 level.

The overall trend of large vascular bundle area and small vascular bundle area of the 2nd and 3rd internodes in each treatment of the two varieties were both decreased from the anthesis to dough stage (Figure 3). Compared with CKR1, large vascular bundle area of the 2nd and 3rd internodes of YN19 in LTR1 and LTR2 significantly decreased by 25.06%, 11.89% and 22.13%, 6.12% at the dough stage, respectively; meanwhile the large vascular bundle area of the 2nd and 3rd internodes of XM26 in LTR1 and LTR2 decreased by 28.28%, 19.54% and 17.56%, 7.19%, respectively. Compared with LTR1, the large vascular bundle area of the 2nd and 3rd internodes of YN19 and XM26 in LTR2 under OPA increased by 17.57%, 20.56% and 12.18%, 12.56%, respectively. At the dough stage in LTR1 and LTR2 treatments, except for the 2nd internode of YN19 and the 3rd internode of XM26, the small vascular bundle area of the 3rd internodes significantly decreased by 12.53% and 11.14% in YN19, and decreased by 20.26% and 6.26% in the 2nd internode of XM26, respectively, compared with CKR1; whereas the small vascular bundle area of the 2nd internodes of XM26 in LTR2 under OPA significantly increased by 17.56%, compared with LTR1. Therefore, spring LTS decreased the large vascular bundle area and small vascular bundle area, while OPA treatment alleviated the damage of spring LTS.

### 2.3. Stem Chemical Composition

#### 2.3.1. Lignin and Cellulose

**Figure 4:**
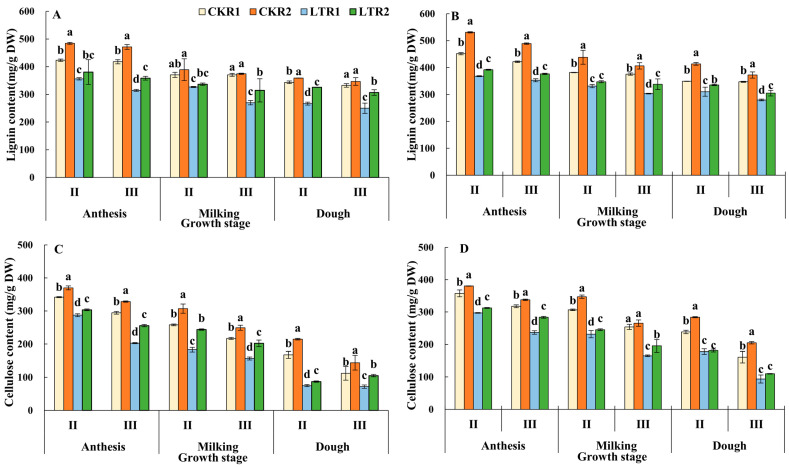
Effects of OPA on stem lignin and cellulose contents of wheat under spring LTS. (**A**) Lignin content of YN19. (**B**) Lignin content of XM26. (**C**) Cellulose content of YN19. (**D**) Cellulose content of XM26. CKR1 and LTR1 represent the 15 °C and −4 °C under traditional phosphorus application, respectively. CKR2 and LTR2 represent the 15 °C and −4 °C under optimized phosphorus application, respectively. II and III indicate the second and third internode of the main stem. Values are mean ± standard deviation. Different lowercase letters above the bars indicate significant differences among treatments for the same cultivar at the 0.05 level.

Following the completion of anthesis, a gradual decline in the lignin and cellulose contents of the 2nd and 3rd internodes was observed in wheat (Figure 4), and this trend remained across different treatments of both varieties. The trend of lignin and cellulose contents at the 2nd and 3rd internodes of the two varieties during anthesis, milking and dough stages is CKR2 > CKR1 > LTR2 > LTR2. At the dough stage, the lignin content of 2nd and 3rd internodes of two varieties in LTR2 was significantly greater than that in the LTR1 treatment; meanwhile, except for the 2nd and 3rd internode of XM26, the cellulose content of 2nd and 3rd internodes of YN19 in LTR2 was significantly higher that of LTR1 treatment. Thus, spring LTS resulted in decreasing lignin and cellulose contents of the 2nd and 3rd internodes; whereas both the contents of lignin and cellulose increased in LTR2 under OPA, compared with the TPA at the same temperature levels, respectively.

#### 2.3.2. Soluble Sugar, Total Nitrogen and C/N Ratio

**Figure 5:**
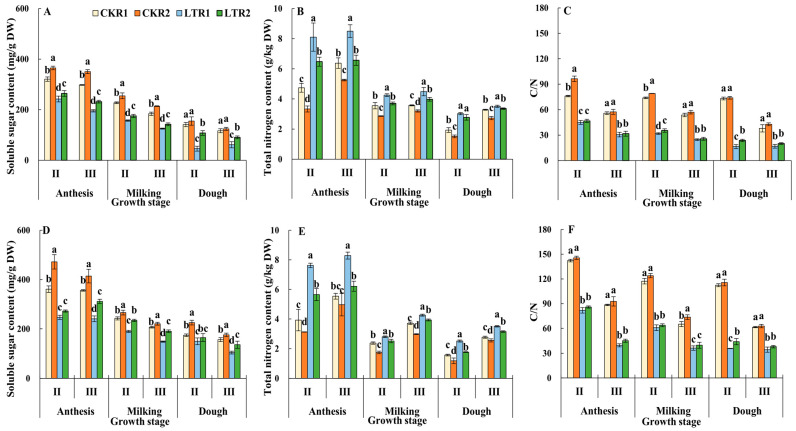
Effects of OPA on the contents of soluble sugar and total nitrogen, and C/N ratio of wheat stem under spring LTS. (**A**) The soluble sugar content of YN19. (**B**) Total nitrogen content of YN19. (**C**) The C/N ratio of YN19. (**D**) The soluble sugar content of XM26. (**E**) Total nitrogen content of XM26. (**F**) The C/N ratio of XM26. CKR1 and LTR1 represent the 15 °C and −4 °C under traditional phosphorus application, respectively. CKR2 and LTR2 represent the 15 °C and −4 °C under optimized phosphorus application, respectively. II and III indicate the second and third internode of the main stem. Values are mean ± standard deviation. Different lowercase letters above the bars indicate significant differences among treatments for the same cultivar at the 0.05 level.

The soluble sugar content, total nitrogen content and the C/N ratio of the 2nd and 3rd internodes showed a decreasing trend in each treatment of the two varieties with the growth of plant (Figure 5). At the dough stage, except for the 2nd internode of XM26, the soluble sugar content of the 2nd and 3rd internodes of two varieties in LTR1 and LTR2 were significantly lower than that of the CKR1 treatment; meanwhile, the change of C/N ratio in all treatments had a similar trend. Additionally, both the soluble sugar content and C/N ratio of the 2nd and 3rd internodes of the two varieties increased in LTR2 under OPA at the dough stage, compared with LTR1, respectively. It is worth noting that spring LTS increased the total nitrogen content of the 2nd and 3rd internodes, and OPA treatment suppressed the increase in total nitrogen content under spring LTS. Thus, the trend of total nitrogen content at the 2nd and 3rd internodes of the two varieties during anthesis, milking and dough stages is LTR1 > LTR2 > CKR1 > CKR2. Therefore, spring LTS decreased the soluble sugar content and C/N ratio, while OPA was beneficial for increasing the soluble sugar content and C/N ratio.

### 2.4. Stem Mechanical Strength and Lodging Resistant Index

**Figure 6:**
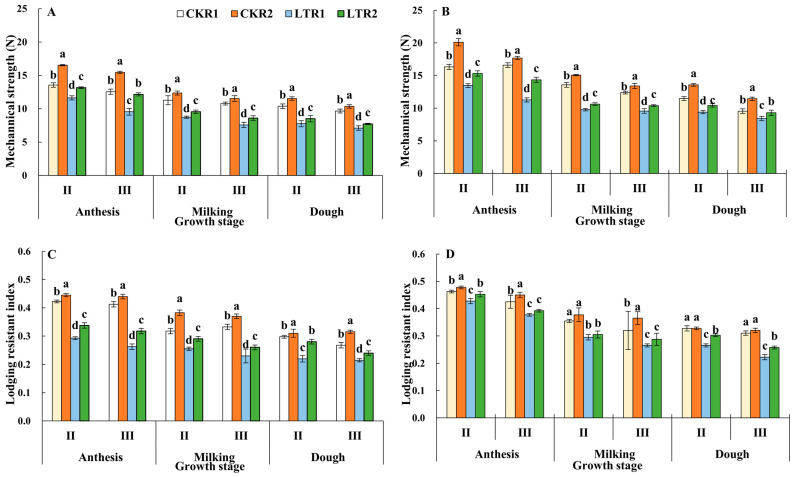
Effects of OPA on the mechanical strength and lodging resistant index of wheat stem under spring LTS. (**A**) Mechanical strength of YN19. (**B**) Mechanical strength of XM26. (**C**) Lodging resistant index of YN19. (**D**) Lodging resistant index of XM26. CKR1 and LTR1 represent the 15 °C and −4 °C under traditional phosphorus application, respectively. CKR2 and LTR2 represent the 15 °C and −4 °C under optimized phosphorus application, respectively. II and III indicate the second and third internode of the main stem. Values are mean ± standard deviation. Different lowercase letters above the bars indicate significant differences among treatments for the same cultivar at the 0.05 level.

As shown in Figure 6, the stem mechanical strength and lodging resistant index of the 2nd and 3rd internodes showed a decreasing trend from the anthesis to dough stage in each treatment of two varieties. At the dough stage, the mechanical strength of 2nd and 3rd internodes of YN19 and XM26 in LTR2 under OPA significantly increased by 9.68%, 8.80% and 10.90%, 10.39% compared with LTR1, respectively; meanwhile, the lodging resistant index of the 2nd and 3rd internodes of YN19 and XM26 in LTR2 under OPA significantly increased by 27.27%, 11.63% and 14.15%, 15.73% compared with LTR1. Thus, spring LTS decreased the mechanical strength and lodging resistant index of the YN19 and XM26, while OPA could alleviate the decrease in stem mechanical strength and lodging resistant index caused by spring LTS.

### 2.5. Grain Yield and Yield Components

Spring LTS decreased the grains per spike, 1000-grain weight and grain yield of the YN19 and XM26, while OPA could alleviate the decrease in grains per spike, 1000-grain weight and grain yield caused by spring LTS (Table 1). Compared with CKR1, the grains per spike of YN19 and XM26 in LTR1 and LTR2 significantly decreased by 15.22%~18.04% and 12.64%~22.57%; whereas the grains per spike of YN19 and XM26 increased by 3.45% and 12.83% in LTR2 under OPA, compared with LTR1, respectively. Meanwhile, the grain yield of YN19 and XM26 in LTR1 and LTR2 treatments significantly decreased by 18.45%~25.73% and 16.75%~28.93%, compared with CKR1, respectively. Additionally, it is worth noting that the grain yield of two varieties under CKR2 treatment was the highest. Therefore, OPA was able to alleviate the yield loss caused by spring LTS, and increased the grain yield without spring LTS.

### 2.6. Correlation Analysis

**Figure 7:**
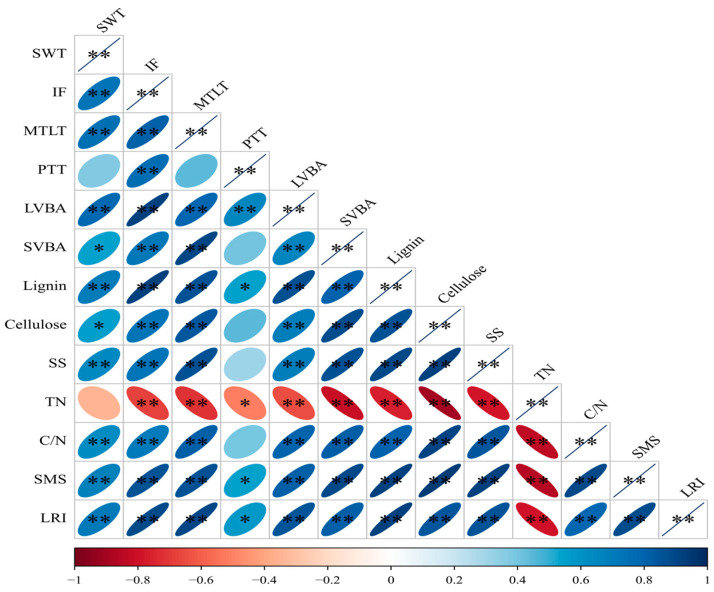
Correlation analysis of stem indexes. * and ** indicate significant differences at the *p* < 0.05 level and *p* < 0.01 level, respectively. SWT, stem wall thickness; IF, internode fullness; MTLT, mechanic tissue layer thickness; PTT, parenchymatous tissue thickness; LVBA, large vascular bundle area; SVBA, small vascular bundle area; SS, soluble sugar; TN, total nitrogen; SMS, stem mechanical strength; LRI, lodging resistant index.

The correlations among stem morphological traits, anatomical parameters, chemical composition, mechanical strength and lodging resistant index at the dough stage are shown in Figure 7. The results showed that the lodging resistant index was significantly and positively related to the SWT, IF, and anatomical parameters like MTLT, PTT, LVBA and SVBA, and the chemical compositions like lignin, cellulose, SS, and the C/N ratio, while it was significantly and negatively correlated with the TN. Stem mechanical strength had a similar correlation with other indexes. Moreover, IF was significantly and positively related to the MTLT, LVBA, SVBA, lignin, cellulose, SS, and the C/N ratio. The results indicated that the lodging resistant index is an integrated trait associated with morphological and anatomical traits, chemical constituents and stem strength.

## 3. Discussion

With the intensification of global climate warming, the growth of winter wheat was accelerated and was more sensitive to spring LTS [9,10,11]. Therefore, spring LTS has become an important limit for the development of high yield, quality and efficiency in wheat production. Many studies have implicated the effects of spring LTS on the function of wheat leaves and the development of spikes [5,14,18,20]. Spring LTS would affect the growth and development of the wheat stem, and may experience elevated risk of stem lodging, ultimately resulting in a significant yield loss in wheat production. However, previous research mostly focused on the source-sink organs, and less consideration of the effect of spring LTS on wheat stem lodging resistance. Wheat lodging resistance is closely related to stem morphology and structure, i.e., plant height, internode fullness, and internode fullness. Muhammad et al. [33] studied the wheat stem characteristics with lodging resistance and found that wall thickness is a key trait that determines stem lodging resistance and is positively correlated with lodging resistance. This study showed that spring LTS decreased the stem wall thickness and internode fullness. This may be due to spring LTS limiting the elongation and development of the internodes, resulting in decreased stem wall thickness and internode fullness. Phosphorus is a major nutrition element and plays a vital role in plant various metabolic activities [23,24]. Optimized phosphorus application could increase nutrition uptake by roots and fulfill the wheat nutrition demand, coordinate the development of source-sink organs and stem assimilate transport, and reduce the risk of stem lodging [31,34,35]. In the present study, the OPA not only increased the stem wall thickness but also increased the internode fullness.

From an anatomical perspective, the lateral growth and development of the stem form the epidermis, thick-walled tissue, vascular bundles, parenchymatous tissue, and medullary cavity from the outside to the inside, respectively [33]. The thickness of stem mechanical tissue could be used as an index to identify the anatomical structure related to lodging resistance. Kong et al. [36] compared the morphological, anatomical and chemical characteristics of four different genotypes of wheat, and found that the mechanical tissue thickness could explain 99% of the lodging resistance changes. The mechanical tissue thickness and vascular bundle area of the wheat stem had a significant correlation with stem strength, and when the mechanical tissue layer thickness is thicker and the vascular bundle area is larger, the stem strength is stronger [37]. In this study, stem mechanic tissue layer thickness, parenchymatous tissue thickness, large vascular bundle area and small vascular bundle area were decreased under spring LTS. However, the thickness of the stem mechanic tissue layer and parenchymatous tissue, and the area of the large vascular bundle and small vascular bundle were increased by the OPA. This may be due to the OPA could alleviate the damage to stem cell wall and promote the growth of stem wall under spring LTS.

Chemical substances in wheat stem participate in the construction of morphological skeleton and the entire process of growth and development, which is the foundation of stem robustness. The stem chemical composition included structural compounds such as cellulose, hemicellulose, lignin, as well as nonstructural compounds such as soluble sugars, all of which could affect stem strength and lodging resistance [38]. Lignin and cellulose are important components of secondary cell walls, which can enhance the mechanical strength of crop stems [39,40]. This study showed that spring LTS decreased the contents of lignin, cellulose, soluble sugar, and the C/N ratio. Xiang et al. [29] found that optimized phosphorus application could significantly increase the content of cellulose and lignin, promote carbohydrate transport in stems and increase stem C/N ratio, and ultimately enhance stem lodging resistance. Similarly, our study also found that OPA increases the content of lignin and cellulose, promotes the accumulation of soluble sugar, and improves the C/N ratio under spring LTS. The results indicate that OPA increases the accumulation of chemical components in wheat stem under spring LTS, especially improving stem C/N ratio.

**Figure 8:**
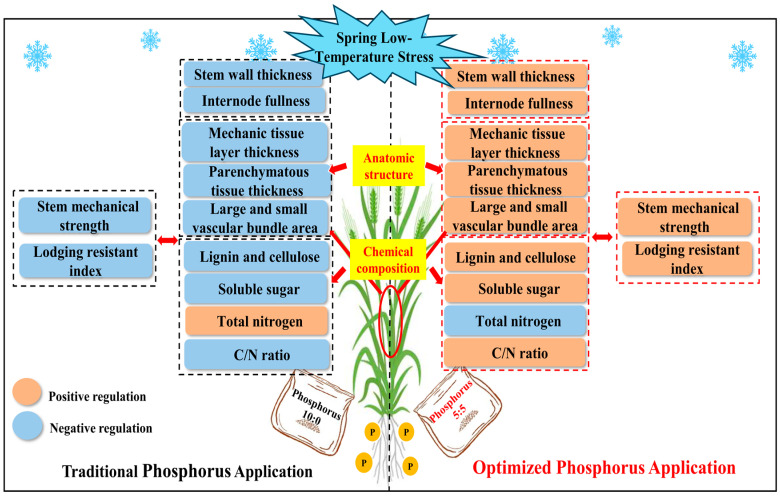
Schematic model in describing the mechanism of wheat stem characteristics under spring LTS by different phosphorus fertilizer treatments. Traditional phosphorus application represents one-off base application at pre-sowing. Optimized phosphorus application represents 50% each at the pre-sowing and jointing stage.

Lodging is an integrated trait associated with many plant characteristics. In addition to morphological and anatomical traits, and chemical constituents, stem strength plays a significant role in stem lodging risk [41,42]. In this experiment, spring LTS decreased the mechanical strength and lodging resistant index of the two varieties, while OPA could alleviate the decrease in stem mechanical strength and lodging resistant index caused by spring LTS. Therefore, the OPA improved stem morphological characteristics, anatomical structure, and chemical compositions of wheat stem, resulting in enhanced mechanical strength and lodging resistance (Figure 8). However, the physiological and molecular mechanisms of OPA improving wheat stem lodging resistance still need to be further studied.

Many previous studies showed that spring LTS could lead to a decrease in wheat yield [5,14,15,20]. The results of this study also indicate that a yield loss in wheat was caused by spring LTS. The OPA could alleviate the decrease in grains per spike, 1000-grain weight and grain yield caused by spring LTS. This may be due to the OPA could promote the nutritional and reproductive growth of wheat, coordinate the competition for assimilates between the source and sink, and improve spikelet development and setting, leading to increased grains per spike and final yield [30,31]. Consequently, the OPA could not only improve the lodging resistance of wheat stems at low temperatures, but also alleviate yield loss. OPA will be a very promising and efficient cultivation measure for wheat stress resistance, which is conducive to the sustainable development of agriculture.

## 4. Materials and Methods

### 4.1. Experiment Design

The experiment was conducted from November 2022 to May 2023 at the Anhui Agricultural University Hefei High Technology Agricultural Garden, Anhui Province, China (31°93′ N, 117°21′ E) during the wheat growing season. Two wheat varieties, Yannong19 (YN19, cold-tolerant variety) and Xinmai26 (XM26, cold-sensitive variety), were used as the test varieties [5]. Wheat varieties were planted in plastic pots (30 cm diameter × 35 cm height; 3 drainage holes). The soil used in the pot experiment was brown soil collected from the 0–20 cm of the top layer. Physicochemical analyses revealed that the collected soil comprised 14.53 g kg^−1^ organic matter, 1.03 g kg^−1^ total nitrogen, 18.25 mg kg^−1^ available phosphorus, 256.02 mg kg^−1^ available potassium and a pH of 5.7. Each pot was filled with 10 kg of sieved soil [43].

Referring to the practice of field fertilization in the experiment region, phosphorus fertilizer was all used as base fertilizers before sowing [30,44]. In this experiment, the mode of phosphorus fertilizer application included traditional phosphorus application (TPA, R1, one-off base application at pre-sowing) and optimized phosphorus application (OPA, R2, 50% each at the pre-sowing and jointing stage) [30,31,32]. The method of temperature levels was divided into normal temperature control (CK, 15 °C) and low-temperature treatment (LT, −4 °C). Four treatments were performed in this experiment, i.e., TPA + CK (CKR1), OPA + CK (CKR2), TPA + LT (LTR1), and OPA + LT (LTR2). Thirty pots were planted in each treatment of two wheat varieties. During the whole wheat growing season, a total of 1.8 g urea (1.2 g of base fertilizer before sowing and 0.6 g of jointing fertilizer) and 1.7 g of potassium sulfate (one-off base application before sowing) were applied to each pot of wheat. The TPA treatment applied 5.0 g of superphosphate (active ingredient percentage ≥ 12.0%) per pot as base fertilizer, while the OPA treatment applied 2.5 g of superphosphate per pot as base fertilizer before sowing and jointing fertilizer, respectively. The top application during the jointing stage was on March 15 in 2023. The low-temperature treatment method was carried out in accordance with Xu et al. [30]. The other cultivation methods, such as pesticide application and irrigation, were conducted following the local optimum cultivation practices to ensure no other stress in these experiments.

### 4.2. Sampling and Measurement

#### 4.2.1. Measurement of Stem Wall Thickness and Internode Fullness

Stem wall thickness and internode fullness of the second and third internode of the main stem were measured at the flowering stage, milking stage and dough stage, respectively. Stem wall thickness was measured using the methods of Zhou et al. [45]. Internode length (L) was measured with a steel rule of 100 cm, then the samples were placed at 105 °C for 30 min and dried to a constant weight at 75 °C, and the internode dry matter mass (M) was determined. Internode Fullness = M/L. Four samples were randomly chosen to measure stem wall thickness and internode fullness for each treatment.

#### 4.2.2. Measurement of Stem Anatomical Parameters

At the flowering stage, milking stage and dough stage, three wheat plants of each treatment with similar size were sampled. The second and third basal internodes of the main stem were chosen to perform paraffin sectioning. The digital section browsing software (CaseViewer 2.4, 3DHISTECH, China) was used for the analysis of mechanic tissue layer thickness, parenchymatous tissue thickness, large vascular bundle area and small vascular bundle area.

#### 4.2.3. Measurement of Stem Chemical Composition

Samples after the determination of internode dry matter mass were thoroughly ground into powder samples. The content of soluble sugar was determined by anthrone colorimetry [46]. Stem sample nitrogen contents were determined by using the H_2_SO_4_-H_2_O_2_ digestion method [47]. The contents of lignin and cellulose were measured using the lignin and cellulose assay kit (Beijing Boxbio Science & Technology Co., Ltd., Beijing, China). The above parameters were measured with three biological replicates. The ratio of C/N was calculated as the following formula:

C/N = Soluble sugar content/Total nitrogen content (1)

#### 4.2.4. Measurement of Stem Mechanical Parameters

Four wheat plants of each treatment with similar sizes were sampled at the anthesis stage, milking stage and dough stage, respectively. Stem mechanical strength of the second and third internode of the main stem was measured using a stem strength tester (YYD-1A, Zhejiang Topu Yunnong Technology Co., Ltd., Hangzhou, Chian) and the center of gravity height was measured with a steel rule of 100 cm according to Zhou et al. [45]. Lodging resistant index was calculated as the following formula:

Lodging resistant index = internode mechanical strength/center of gravity height(2)

#### 4.2.5. Yield Components and Grain Yield

At the maturity stage, three main stems of wheat plants were randomly selected from each treatment to investigate the grain number per spike, 1000-grain weight and grain yield per stem.

### 4.3. Statistical Analysis

Microsoft Excel (version 2019; Microsoft, Inc., Redmond, WA, USA) was used for data sorting and figure and table production. Statistical analyses were performed using SPSS 19.0 software (IBM SPSS 19.0, SPSS Inc., Chicago, IL, USA). The significant differences in measurement parameters among the treatments were identified by Duncan’s multiple comparison tests (*p* < 0.05) via one-way ANOVA. The correlations analysis was plotted according to Pearson’s method by using RStudio.

## 5. Conclusions

Spring LTS decreased the stem wall thickness and internode fullness, and altered stem anatomical structure and chemical composition, resulting in a decrease of wheat stem mechanical strength and lodging resistant index. Optimization of phosphorus application was able to the regulate stem characteristics. The results of our study showed that OPA could increase the stem wall thickness and internode fullness under spring LTS. OPA could increase stem mechanic tissue layer thickness, parenchymatous tissue thickness, large vascular bundle area and small vascular bundle area, and alleviate the damage to stem cell walls caused by spring LTS. Simultaneously, OPA increased the contents of lignin, cellulose, and soluble sugar, and improved the C/N ratio, thereby augmenting the stem mechanical strength and lodging resistant index under spring LTS. Notably, OPA was able to alleviate the yield loss caused by spring LTS, and increased the grain yield under normal temperature conditions. Therefore, optimized phosphorus application (50% each at the pre-sowing and jointing stage) would be meaningful and practical for improving crop resistance to stress, especially in the interest of sustainable agricultural development.

## Figures and Tables

**Table 1:** OPA affects grain yield and its composition under spring LTS.

Variety	Treatment	Grains per Spike	1000-Grain Weight (g)	Grain Yield (g/stem)
YN19	CKR1	46.0 ± 2.0 a	44.80 ± 1.69 a	2.06 ± 0.07 a
	CKR2	48.0 ± 1.0 a	44.97 ± 0.54 a	2.16 ± 0.06 a
	LTR1	37.7 ± 1.5 b	40.56 ± 0.81 b	1.53 ± 0.01 c
	LTR2	39.0 ± 2.0 b	43.26 ± 2.11 ab	1.68 ± 0.01 b
XM26	CKR1	44.3 ± 2.1 b	44.34 ± 1.73 ab	1.97 ± 0.14 b
	CKR2	49.7 ± 2.9 a	46.56 ± 2.70 a	2.32 ± 0.26 a
	LTR1	34.3 ± 1.5 d	40.88 ± 2.48 b	1.40 ± 0.03 c
	LTR2	38.7 ± 1.5 c	42.62 ± 3.61 ab	1.64 ± 0.09 c

Note: CKR1 and LTR1 represent the 15 °C and −4 °C under traditional phosphorus application, respectively. CKR2 and LTR2 represent the 15 °C and −4 °C under optimized phosphorus application, respectively. Values are mean ± standard deviation. Different lowercase letters above the bars indicate significant differences among treatments for the same cultivar at the 0.05 level.

## Data Availability

Data is contained within the article.

## References

[1] Mao H., Jiang C., Tang C., Nie X., Du L., Liu Y., Cheng P., Wu Y., Liu H., Kang Z. (2023). Wheat adaptation to environmental stresses under climate change: Molecular basis and genetic improvement. Mol. Plant.

[2] Announcement by the National Bureau of Statistics on Grain Production Data for 2023. https://www.stats.gov.cn/sj/zxfb/202312/t20231211_1945417.html.

[3] Ma Z.Y., Ren J.X., Chen H.T., Jiang H., Gao Q.X., Liu S.L., Yan W., Li Z.M. (2022). Analysis and recommendations of IPCC working groupⅠassessment report. Res. Environ. Sci..

[4] Ye L., Wang T., Wu R., Zheng C., Zhan L., Chen J., Guo S., Chen Y. (2024). Evaluation of cold resistance at seedling stage for 70 peanut genotypes based on photosynthetic fluorescence characteristics. Agronomy.

[5] Zhang Y., Cai H., Liu L., Xu H., Chen X., Li J. (2023). Screening of varieties resistant to late-spring coldness in wheat and effects of late-spring coldness on the ultrastructure of wheat cells. Agronomy.

[6] Crimp S.J., Zheng B.Y., Khimashia N., Gobbett D.L., Scott Chapman S., Howden M., Nicholls N. (2016). Recent changes in southern Australian frost occurrence: Implications for wheat production risk. Crop Pasture Sci..

[7] Trnka M., Rötter R., Ruiz-Ramos M., Kersebaum K.C., Olesen J.E., Zalud Z., Semenov M.A. (2014). Adverse weather conditions for European wheat production will become more frequent with climate change. Nat. Clim. Change.

[8] Holman J.D., Schlegel A.J., Thompson C.R., Lingenfelser J.E. (2011). Influence of precipitation, temperature and 56 years on winter wheat yields in western Kansas. Crop Manag..

[9] Hao Z.X., Geng X., Wang F., Zheng J.Y. (2018). Impacts of climate change on agrometeorological indices at winter wheat overwintering stage in northern China during 2021–2050. Int. J. Climatol..

[10] Jiang G., Hassan M.A., Muhammad N., Arshad M., Chen X., Xu Y.H., Xu H., Ni Q.Q., Liu B.B., Yang W.K. (2022). Comparative physiology and transcriptome analysis of young spikes in response to late spring coldness in wheat (*Triticum aestivum* L.). Front. Plant Sci..

[11] Li X., Jiang D., Liu F. (2016). Winter soil warming exacerbates the impacts of spring low temperature stress on wheat. J. Agron. Crop Sci..

[12] Xiao L.J., Liu L.L., Senthold A., Xia Y.M., Tang L., Liu B., Cao W.X., Zhu Y. (2018). Estimating spring frost and its impact on yield across winter wheat in China. Agric. For. Meteorol..

[13] Zhang Y., Ni C., Dong Y., Jiang X., Liu C., Wang W., Zhao C., Li G., Xu K., Huo Z. (2023). The role of the ascorbic acid–glutathione cycle in young wheat ears’ response to spring freezing stress. Plants.

[14] Zhang Y., Liu L.Z., Chen X., Li J.C. (2022). Effects of low-temperature stress during the anther differentiation period on winter wheat photosynthetic performance and spike-setting characteristics. Plants.

[15] Chen X., Liu L.L., Cai H.M., Zheng B.Q., Li J.C. (2024). Effects of spring low-temperature stress on winter wheat seed-setting characteristics of spike. Plant Soil. Environ..

[16] Hu X.Y., Ma J.F., Qian W.H., Cao Y., Zhang Y., Liu B., Tang L., Cao W.X., Zhu Y., Liu L.L. (2022). Effects of low temperature on the amino acid composition of wheat grains. Agronomy.

[17] Shi K.J., Yin T.W., Zhu Y., Liu B., Tang L., Cao W.X., Liu L.L. (2022). Estimating the effect of low-temperature stress on the spatial distribution patterns of protein in wheat grains. J. Cereal Sci..

[18] Liu L.Z., Zhang Y., Zhang L., Cai H.M., Yu M., Wei F.Z., Chen X., Li J.C. (2023). Effects of low temperature stress during the anther differentiation period on leaf anatomical structure and photosynthetic characteristics of wheat. Chin. J. Agrometeorol..

[19] Ke Y.Y., Chen X., Zhang L.L., Zhang Y., Xu H., Hassan M.A., Jiang G., Lin F.F., Wei F.Z., Li J.C. (2021). Effects of low temperature stress at anther connective stage on dry matter accumulation, translocation and distribution and grain yield of wheat. J. Anhui Agric. Univ..

[20] Lin F.F., Li C., Xu B., Chen J., Chen A.H., Hassan M.A., Liu B.B., Xu H., Chen X., Sun J.Q. (2023). Late spring cold reduces grain number at various spike positions by regulating spike growth and assimilate distribution in winter wheat. Crop J..

[21] Zhang L., Chen X., Li J.C. (2022). Bibliometric analysis of Chinese wheat lodging research literature based on CNKI. J. Jianghan Univ. (Nat. Sci. Ed.).

[22] Technical Opinions on Promoting Recovery and Reducing Losses of Lodging Wheat. http://www.moa.gov.cn/gk/nszd_1/nszd_2/202405/t20240516_6455626.htm.

[23] Poirier Y., Jaskolowski A., Clua J. (2022). Phosphate acquisition and metabolism in plants. Curr. Biol..

[24] Yaakob M.A., Mohamed R.M.S.R., Al-Gheethi A., Ravishankar G.A., Ambati R.R. (2021). Influence of nitrogen and phosphorus on microalgal growth, biomass, lipid, and fatty acid production: An overview. Cells.

[25] Alewell C., Ringeval B., Ballabio C., Robinson D.A., Panagos P., Borrelli P. (2020). Global phosphorus shortage will be aggravated by soil erosion. Nat. Commun..

[26] Xu H., Hassan M.A., Sun D., Wu Z., Jiang G., Liu B., Ni Q., Yang W., Fang H., Li J.C. (2022). Effects of low temperature stress on source–sink organs in wheat and phosphorus mitigation strategies. Front. Plant Sci..

[27] Deng Y., Zhao L., Anwar S., Zhang L.G., Shafiq F., Guo H.X., Qin L.X., Wang M.X., Wang C.Y. (2022). Phosphorus fertigation conferred lodging tolerance and improved grain quality in *Chenopodium quinoa* via enhanced root proliferation and stalk strength. J. Soil. Sci. Plant Nutr..

[28] Liang Z.Y., Xue J., Zhang G.Q., Min B., Shen D.P., Fang L., Zhou L.L., Zhang Y.Q., Yang H.S., Wang K.R. (2023). Effects of phosphorus application rate on lodging resistance of maize under integrated water and fertilizer. Crops.

[29] Xiang D.B., Guo K., Lei T., Yu X.B., Luo Q.M., Yang W.Y. (2010). Effect of phosphorus and potassium on stem characteristic and lodging resistance of relay cropping soybean. Chin. J. Oil Crop Sci..

[30] Xu H., Hou K.Y., Fang H., Liu Q.Q., Wu Q., Lin F.F., Deng R., Zhang L.J., Chen X., Li J.C. (2023). Twice-split phosphorus application alleviates low-temperature impacts on wheat by improved spikelet development and setting. J. Integr. Agric..

[31] Xu H., Wu Z., Xu B., Sun D., Hassan M.A., Cai H., Wu Y., Yu M., Chen A., Li J. (2022). Optimized phosphorus application alleviated adverse effects of short-term low-temperature stress in winter wheat by enhancing photosynthesis and improved accumulation and partitioning of dry matter. Agronomy.

[32] Xu H., Hassan M.A., Li J.C. (2023). Twice-split phosphorus application alleviates low temperature stress by improving root physiology and phosphorus accumulation, translocation, and partitioning in wheat. Agronomy.

[33] Muhammad A., Hao H.H., Xue Y.L., Alam A., Bai S.M., Hu W.C., Sajid M., Hu Z., Samad R.A., Li Z.H. (2020). Survey of wheat straw stem characteristics for enhanced resistance to lodging. Cellulose.

[34] Shen J.B., Yuan L.X., Zhang J.L., Li H.G., Bai Z.H., Chen X.P., Zhang W.F., Zhang F.S. (2011). Phosphorus dynamics: From soil to plant. Plant Physiol..

[35] Jahan M., Amiri M.B. (2018). Optimizing application rate of nitrogen, phosphorus and cattle manure in wheat production: An approach to determine optimum scenario using response-surface methodology. J. Soil. Sci. Plant Nutr..

[36] Kong E.Y., Liu D.C., Guo X.L., Yang W.L., Sun J.Z., Li X., Zhan K.H., Cui D.Q., Lin J.X., Zhang A.M. (2013). Anatomical and chemical characteristics associated with lodging resistance in wheat. Crop J..

[37] He J., Sun S.G., Ge C.B., Song D.Y., Qiao J.L., Li S.P., Su Y.R., Liao P.G. (2022). Relationship between microstructure, biochemical composition and stem strength of different wheat varieties (lines). Acta Agric. Boreali-Sin..

[38] Zhao X., Zhou S.L. (2022). Research progress on traits and assessment methods of stalk lodging resistance in maize. Acta Agron. Sin..

[39] Ma J.F., Yamaji N. (2006). Silicon uptake and accumulation in higher plants. Trends Plant Sci..

[40] Shah A.N., Tanveer M., Rehman A.U., Anjum S.A., Iqbal J., Ahmad R. (2017). Lodging stress in cereal-effects and management: An overview. Environ. Sci. Pollut. Res..

[41] Berry P.M., Stering M., Spink J.H., Baker C.J., Sylvester-Bradley R., Mooney S.J., Tams A.R., Ennos A.R. (2004). Understanding and reducing lodging in cereals. Adv. Agron..

[42] Berry P.M., Sylvester-Bradley R., Berry S. (2007). Ideotype design for lodging-resistant wheat. Euphytica.

[43] Huang W.X., Dai W.C., Chen T.T., Cai H.M., Weng Y., Tang Z.W., Yin C., Wang P.N., Zheng B.Q., Li J.C. (2024). Spraying KH_2_PO_4_ enhances the physiological activity of wheat flag leaves and roots to alleviate the damage caused by late spring coldness. Plant Nutr. Fert. Sci..

[44] He J.N., Shi Y., Yu Z.W. (2019). Subsoiling improves soil physical and microbial properties, and increases yield of winter wheat in the Huang-Huai-Hai Plain of China. Soil. Tillage Res..

[45] Zhou J., Wang X., Zhu Y.L., Liu H.H., Chen X., Wei F.Z., Sun J.Q., Song Y.H., Li J. (2019). Effect of nitrogen fertilizer management on stem lodging resistance and yield of wheat. J. Triticeae Crop..

[46] Wang X., Wu Z., Zhou Q., Wang X., Song S., Dong S. (2022). Physiological response of soybean plants to water deficit. Front. Plant Sci..

[47] Ni Q.Q., Cai H.M., Wu H., Jiang G., Xu B., Wei F.Z., Chen X., Li J.C. (2023). Effect of straw returning in different seasons on dry matter accumulation, distribution and nitrogen use efficiency of winter wheat. J. Triticeae Crop..

